# Effect of widowhood on the risk of disability among the elderly in China

**DOI:** 10.3389/fpsyt.2023.1169952

**Published:** 2023-05-18

**Authors:** Jiahui Pang, Shouyi Xu, Yuanyang Wu

**Affiliations:** ^1^School of Public Administration, Zhongnan University of Economics and Law, Wuhan, China; ^2^School of Medicine and Health Management, Tongji Medical School, Huazhong University of Science and Technology, Wuhan, Hubei, China

**Keywords:** widowhood, disability, healthy aging, individual and time fixed effect, pressure process model, marriage resource theory

## Abstract

**Background:**

The deteriorating health status of widowed elderly individuals becomes an important restriction factor affecting healthy aging in China. This paper aimed to find effective ways to reduce the risk of disability among the widowed elderly.

**Methods:**

An empirical analysis was conducted by using four surveys from the China Health and Retirement Longitudinal Study (CHARLS) in 2011, 2013, 2015, and 2018. A fixed-effects model was performed to estimate the effect of widowhood on the disability risk of the elderly in China and its disparities in different groups, and influencing channels and moderating effects were further investigated.

**Results:**

Widowhood significantly increased the risk of disability, and the results were robust. The risk of disability was higher among those who were male, living in urban areas, educated, and 60-to-70-years-old. Possible channels in the association were psychological stress and unhealthy behavior. Meanwhile, more financial support, contact from children, and social activities decreased the risk of disability.

**Conclusion:**

The health risks of older adults after widowhood can be reduced by concerted efforts from society and government, including promoting the traditional Chinese virtue of filial piety and providing health interventions and social support services.

## Introduction

Based on China’s large population, aging population, and family patterns, we aimed to explore the health issues of the elderly widowed population in China. Currently in China, the development of medical technology can extend the average life span, but an extended life expectancy is not the same as a healthy life expectancy, and disability is a common survival state in later life. In the characteristic Chinese family model, spouses play the roles of resource sharing, spiritual companionship, and health supervision. With the deepening of aging in China, there is a growing group of widowed elderly in China, and thus also the disappearance of positive health externalities from spouses after a widowhood event. Therefore, it is urgent to explore whether widowhood affects the risk of incapacity and it is meaningful to find ways to reduce the risk of incapacity in the widowed group in later life.

On the one hand, the development of medical technology has reduced the risk of death. Lives can be sustained, but a longer life span does not mean a longer healthy life span due to the increasing probability of surviving with disease in older age groups (1). In terms of disability, the number of moderately and severely disabled elderly people aged over 65 years is expected to be up to 83.04 million in 2050 (2), and the proportion of time spent with moderate and severe disability in the remaining life expectancy is also expected to rise rapidly (3). Generally, health depreciation is higher in older age groups, especially the widowed elderly people who face dilemmas from the resource constraints and psychological pressure which make the widowed elderly a vulnerable group in the context of healthy aging. On the other hand, marriage is a sign of the existence and union of a family, and widowhood breaks the union and family structure, which has an impact on individual health. From the perspective of family care, with the development of the nuclear family and small family, as for the care needs of the elderly group, spouses can not only provide life care and spiritual comfort, but also reduce the risk of accidents. Being widowed means a reduction in physical care, emotional comfort, financial support, and qualitied life. However, the proportion of older people with a spouse in China is not optimistic, and the total number of widowed elderly people is expected to grow to 118.4 million in 2050 (4). A focus on widowhood, especially on the effects of widowhood on health in later life, is not only a need for general health promotion, but also a requirement for scientific answers to questions about its impact outcomes, impact channels, population disparities, and for deepening the development of population theory.

Marital status has a significant impact on the health of the elderly group. Marriage positively influences both the health level and survival life span of individuals (5). According to data, marriage is the factor that most affects the healthy life expectancy of China’s elderly population, with an impact of about 60% (6). Firstly, based on the marriage resource theory, marriage has a health-protective effect, curbing health deterioration (7) and influencing the health behavior of older adults (8). With a living spouse, the spouse positively influences both health behavior management and the health management environment of older adults (9). Married groups are more likely to have healthy lifestyles, good dietary habits, and do more exercise (10, 11). For example, marriage controls the tendency of individual health behaviors in older adults through social integration, and the sense of obligation and responsibility generated by marriage can offset negative health behaviors, thus positively influencing the health of older adults (8).

Secondly, the stress process model suggests that, with increased age, the frequent death of relatives becomes an important stressor (12) gradually, and being in a stress-exposed environment or having a lack of resources can have adverse effects on an individual’s physiological and psychological health (13). For widowed elderly individuals, on the other hand, health behaviors due to a lack of social connections can lead to decreased health status (2, 14). Studies have found that older adults who are married have a lower risk of death, a longer average remaining life expectancy, and a relatively longer expected time to complete self-care (3). Having a spouse can significantly reduce the health burden of older adults, especially when later life care is required, and the health support that a spouse brings increases with the level of frailty of the individual (15). When spouses are unable to provide care, older adults who are widowed have higher rates of depressive tendencies or depression (16) and lower life satisfaction, which leads to the increased risk of death (17). Additionally, widowhood has a negative impact on health in later life. For example, psychological stress becomes worse in older adults after widowhood (14), with significantly higher rates of Instrumental Activity of Daily Living disability (IADL disability) in widowed older adults (18), and reduced quality of life due to widowhood (19). Finally, in the context of current family marriage patterns in China, based on data from previous national censuses, the proportion staying lifetime single is still very low, with less than 1% of women and less than 4% of men remaining unmarried over their lifetime in China.

Previous research into the health effects of widowhood events have focused on the following three dimensions: physical health (20–23), mental health (22, 24–29), and mortality (30–32). In the physical health dimension, the academic community have generally used self-rated health indicators (20, 21, 23) and objective health indicators such as activity of daily living, cognitive ability (20), number of chronic diseases (24), and sleeping time (21). It is common practice to select multiple indicators for different dimensions of health measurement. Systematic research on the disability risk needs to be expanded, and further research is needed on whether the absence of a spouse functioning as the primary provider of family care worsens the ability of older adults to live, and how to avoid the health problems that follow from the absence of spousal care.

Based on the above, this paper focuses on the impact of bereavement on the disability risk in elderly groups. Based on the stress process theory, we selected data from the China Health and Retirement Longitudinal Study (CHARLS) over four periods from 2011 to 2018. We then analyzed the impact of widowhood events on the disability risk of the elderly in later life and its heterogeneity among different groups using the individual and time fixed effect model, before investigating its impact channels and moderation. The purpose of this paper was to find effective ways to reduce the risk of disability in the late-life widowed group and thus protect their health.

## Methods

### Data sources

Data for this article are from the 2011, 2013, 2015, and 2018 China Health and Retirement Longitudinal Study (CHARLS). Organized by Peking University, the CHARLS data are based on China’s middle-aged and elderly population, and the national baseline survey was conducted in 2011, with a sample size covering 28 provinces (autonomous regions and municipalities directly under the Central Government), 150 counties, 450 communities (villages), and 12,400 households by the time the national follow-up survey was completed in 2018. Our research design is a longitudinal study, but each survey will not only track previous respondents but also include new respondents. In the four periods of data used in this paper, there are single-period respondents as well as multi-period respondents, and there is no exclusion of respondents who lose access over time. The criterion for missing values in this paper is the presence of missing values in the relevant data of the sample. This paper first removed samples under the age of 60, followed by removing samples with missing values, resulting in a final sample size of 7,986.

### Key variables

#### Explained variables

The explanatory variable in this paper is the risk of disability, as measured by activities of daily living (ADL). The Katz index is generally used regarding the definition of disability, which determines the degree of disability of the elderly by measuring their independence in completing six daily activities: getting up, eating, dressing, bathing, urinating and defecating, and indoor activities. If one or more of the above activities cannot be completed independently or are limited, the elderly person is considered to be disabled and is assigned the value of 1; if the elderly person completes all the above activities independently, the value is assigned 0.

#### Explanatory variables

In this paper, two types of marital status, widowed and married with spouse present, were selected as the core explanatory variables, with married with spouse present assigned the value of 0 and widowed assigned the value of 1, generating a dummy variable for widowhood.

#### Control variables

Age, age squared, gender, household registration, years of education, the number of chronic diseases, whether living with children, the number of children, and the log of personal assets were selected as the control variables in this paper, where gender, household registration^1^ and whether living with children are dummy variables; specifically, females were assigned the value of 1 and males the value of 0; urban areas were assigned the value of 1 and rural areas the value of 0; living with children was assigned the value of 1 and others are assigned the value of 0. Age, age squared, years of education, the number of chronic diseases, the number of children, and the log of personal assets were continuous variables.

#### Channel variables

The impact of widowhood on the risk of disability in elderly individuals may occur through three possible channels: psychological stress, health behavior, and future health expectations. Channel 1: widowhood generates psychological stress and further influences physiological health; channel 2: the widowed elderly cope with stress through unhealthy life behaviors; channel 3: health is the consumer good and the investment good, and the stress caused by widowhood influences the widowed elderly’s future health expectations and changes their health investment behavior, which in turn affects their long-term health. In this paper, we use sleep duration, smoking, and personal fitness expenditure^2^ as the indicators to explore the Channels.

#### Moderating variables

Children’s financial support, frequency of children’s contact, and the number of social activities were selected as the moderating variables to investigate whether child care and social participation could mitigate the stress exposure and resource loss caused by widowhood. The questions “In the past year, how much financial support did you or your spouse receive from your children when they were not living with you?,” “How often do you communicate with your children by phone, text message, WeChat, letter, or email when you are not living with your child?,” and “Have you done any of the following social activities in the past month?” from the CHARLS questionnaire were used to produce the data for children’s financial support, frequency of children’s contact, and the number of social activities as the continuous variables, respectively.

### Models

#### Individual and time fixed effect model

A widowhood event can be considered an exogenous shock, but there may still be an endogeneity problem regarding omitted variables. In terms of model setting, the Hausman test revealed (*p* < 0.01) that the fixed effects model is more appropriate for analysis. Based on the theoretical analysis and the characteristics of the data used in this paper, the individual and time fixed effect model was constructed as the following:


(1) $$Katz\_dummy_{it}=\beta Marital\_status_{it}+\gamma x_{it}+\lambda_{i}+\delta_{t}+\mu_{it}$$

where $Katz\_dummy_{it}$ is the disability status of the sample i at the time t , $Marital\_status_{it}$ is the marital status of the sample i at the time t , $x_{it}$ is the control variable consisting of age, age squared, gender, household registration, education level, the number of chronic diseases, whether living with children, the number of children, and the log of personal assets of the sample i at the time t , $\lambda_{i}$ represents individual fixed effects, $\delta_{t}$ represents time fixed effects, and $\mu_{it}$ is a random error term.

#### Channel analysis

Based on the fact that causal relationships in the phenomena of human socioeconomic life are often intertwined and intricate, when the causal relationship from a specific cause to a specific outcome is initially verified by data, it is necessary to explore which intermediate variables in the causal chain affected the outcome because the causal relationship between phenomena may contain multiple logical links and the cause does not act directly on the outcome (32). The purpose of the channel analysis in this paper is to further explore the cause-and-effect relationships. When widowhood increasing the risk of disability was proven by data, it was further explored to investigate whether sleep duration, smoking, and personal fitness expenditure were channels in the causal relationship. There have been many studies using channel analysis (33–34), and the channel analysis section of this paper drew on the common practice of channel analysis, where in the first step, the causality between channel variables and explanatory variables was theoretically more intuitive and closer in terms of logical and spatiotemporal relationships so that it was not necessary to use formal causal inference instruments to study the causal relationship from the channel variable to the explanatory variable, and the second step only looked at the effect of the explanatory variable on the channel variable (32).

In exploring the channel of the effect of widowhood on the risk of disability, the explained variables in Equation (1) were replaced with three channel variables: sleep duration, smoking, and personal fitness expenditure, referring to existing studies (19, 33). The model was set as follows:


(2) $$Mechanism_{it}=\beta_{2}Marital\_status_{it}+\gamma x_{it}+\lambda_{i}+\delta_{t}+\mu_{it}$$

where $Mechanism_{it}$ were the channel variables of interest in this study, including sleep duration, smoking, and personal fitness expenditure. If $\beta_{2}$ was significant, we considered it as a channel variable. The other variables were defined in the same way as in Equation (1).

#### Moderating effect model

Considering that the effects of widowhood may be moderated by family or social support, an interaction term is added here to explore the moderating effects of family children’s financial support, family children’s spiritual comfort, and external social support factors on widowhood. The specific model was set as the following:


(3) $$\begin{array}{l} Katz\_dummy_{it}=\beta_{1}Marital\_status_{it}*\mathrm{mod}erating_{it}\\ \;\;\;\;\;\;\;\;\;\;\;\;\;\;\;\;\;\;\;\;\;\;\;\;\;\;\;\;\;\;\;\;\;\;\;\;\;\;\;\;\;\;\;\;+\gamma x_{it}+\lambda_{i}+\delta_{t}+\mu_{it} \end{array}$$

where $\mathrm{mod}erating_{it}$ is the family children’s financial support, family children’s spiritual comfort, and external social support in period t for the sample i .

## Results

### Descriptive analysis of variables

Table 1 shows the basic descriptive statistics of each variable. In terms of risk of disability, about 8.7% of the population is disabled with the marital status of married with spouse present, and about 15.4% with the marital status of widowhood; it is tentatively inferred that the risk of disability of the elderly who are married with spouse present may be lower compared with the elderly who are widowed. From the perspective of marital status, about 41.16% of the elderly are in the state of widowhood, and about 58.84% of the elderly are married with spouse present. Although the proportion of widowhood is lower than that of the married with spouse present group, it is not a small group, and the proportion of the widowed group will gradually increase as age rises. Regarding age, the average age of the widowed group is higher than that of the married with spouse present elderly group by about 6 years. From the perspective of gender, the proportion of female elderly in the married with spouse present group is about 64.7%, and the proportion of female elderly in the widowed group is as high as about 83.7%. The proportion of females in the widowed group is higher than that of the married with spouse present group by about 19%, which is consistent with the reality that elderly males have a shorter life expectancy. The overall sample of elderly had a short year of education, one or more chronic diseases on average, three or more children on average, slept for about six hours, and did not have a high proportion of smokers. The widowed group had a higher proportion of rural households, shorter years of education, a higher proportion of living with children, fewer personal assets, more personal fitness expenditure, less financial support from children, more frequent contact from children, and a higher number of social activities compared to the married with spouse present group.

**Table 1:** Basic descriptive statistics.

Variable	Married with spouse present		Widowhood		Mean-diff	*t*
	*N*	Mean	*N*	Mean		
Disability risk	4,699	0.087	3,287	0.154	−0.067***	−9.282
Age	4,699	67.464	3,287	73.224	−5.760***	−37.628
Age squared	4,699	4588.698	3,287	5442.882	−854.184***	−37.463
Gender	4,699	0.647	3,287	0.837	−0.190***	−19.142
Household registration	4,699	0.244	3,287	0.202	0.042***	4.392
Years of education	4,699	5.274	3,287	3.433	1.841***	18.185
Number of chronic diseases	4,699	1.375	3,287	1.373	0.003	0.085
Whether living with children	4,699	0.499	3,287	0.646	−0.147***	−13.146
Number of children	4,699	3.384	3,287	3.963	−0.580***	−15.300
Log of personal assets	4,699	6.127	3,287	4.502	1.624***	21.062
Sleep duration	4,699	5.987	3,287	5.811	0.176***	3.496
Whether smoking	4,699	0.094	3,287	0.100	−0.006	−0.865
Personal fitness expenditure	4,699	82.866	3,287	124.051	−41.184***	−3.970
Financial support from children	4,699	2525.733	3,287	2372.522	153.211	0.995
Frequency of child contact	4,699	6.531	3,287	8.018	−1.488***	−8.173
Number of social activities	4,699	0.797	3,287	0.859	−0.062***	−2.926

*p < 0.10, **p < 0.05, ***p < 0.01.

### Basic regression results

Since regression using the logit panel data model censors a large number of individuals in the group without variation, panel least squares regression was also performed to ensure data adequacy, and the results remained consistent. Table 2 shows the regression results of widowhood on the risk of disability among the elderly, where Model 1 and Model 2 are OLS regression results, and Model 3 and Model 4 are logit regression results. The regression results consistently indicated that the risk of disability is significantly higher for the widowed elderly compared to the elderly married with spouse present, and there is a significant positive relationship between widowhood and the risk of disability among elderly individuals.

**Table 2:** Effect of widowhood on the elderly’s risk of disability.

Variable	OLS		Logit	
	(1)	(2)	(3)	(4)
	Disability risk	Disability risk	Disability risk	Disability risk
Widowhood	0.090***	0.055**	1.042***	0.693*
	(0.012)	(0.024)	(0.150)	(0.358)
Control variables	YES	YES	YES	YES
Constant	0.100***	1.556***		
	(0.003)	(0.337)		
*N*	33,084	11,772	6,247	1,360
*R* ^2^	0.003	0.049		

*p < 0.10, **p < 0.05, ***p < 0.01.

In addition, other variables influenced the risk of disability among the elderly to varying degrees. There was a significant and U-shaped relationship between age and risk of disability, with the risk of disability decreasing with age until about 90.9 years of age and increasing with age after 90.9 years of age. In old age, age gradually becomes an important factor affecting individual disability, which is consistent with the trend of disability probability in each age group from 2013 to 2018 (35). At the same time, it was found that the risk of disability is higher in the female elderly group than in the male group, which may stem from: firstly, health deprivation caused by early childbearing affecting female health in later life; secondly, the fact that females live longer on average and thus older females are more likely to become widowed, and the risk of disability increases along with longer life span; and/or thirdly, compared with males, most females give more time and energy to the care of their spouses and children in the family, and their caregiver role in the family often involves more physical and mental stress and health attrition. Chronic diseases and the complications of chronic diseases increase the health risks of individuals; as the number of chronic diseases increases, the higher risk of disability among the elderly may be due to the high level of physical health impairment caused by chronic diseases. Living with children and having better personal assets can reduce the risk of disability among the elderly. Children can bring financial support, physical care, and spiritual companionship to the elderly, and better financial status increases the availability of resources for the elderly.

### Robustness test

To further verify the above findings, robustness tests were conducted by replacing the data sample, the disablement measurement index, and the analysis method in three ways. The data were selected from the Chinese Longitudinal Healthy Longevity Survey (CLHLS) database in 2018, and the Barthel index in the activity of daily living scale was used to explore the effect of widowhood on the risk of disability among the elderly. Notably, since the 2018 CLHLS data were cross-section data, propensity score matching (PSM) was used here to overcome the sample selection bias, and the matched sample was then used for regression analysis. The regression results are presented in Table 3, with Model (5) and Model (6) for the Katz index as a measure, and Model (7) and Model (8) for the Barthel index as a measure of disability. The regression results were all consistent with the previous statement that widowed marital status significantly increases the risk of disability in the elderly group compared to being married with the spouse present.

**Table 3:** Robustness analysis.

	(5)	(6)	(7)	(8)
	Disability risk (KATZ)	Disability risk (KATZ)	Disability risk (Barthel)	Disability risk (Barthel)
Widowhood	0.046**	0.047***	−2.809***	−2.873***
	(0.018)	(0.017)	(0.984)	(0.845)
Control variable	Yes	Yes	Yes	Yes
Constant	0.211***	1.073*	84.806***	47.869*
	(0.013)	(0.556)	(0.695)	(27.822)
*N*	2,162	2,162	2,162	2,162
*R* ^2^	0.003	0.143	0.004	0.269

*p < 0.10, **p < 0.05, ***p < 0.01.

### Disparities analysis

The effect of widowhood on the risk of disability among the elderly may be influenced by individual characteristics and social and economic factors, thus showing differences. Therefore, four dimensions of gender, urban–rural, education level, and age were selected for subsample regression to explore the heterogeneity of the effect of widowhood on the risk of disability among the elderly, and the results are shown in Tables 4, 5. Compared with married with spouse present, widowhood significantly increased the risk of disability among male, urban, highly educated, and 60–70 years old cohorts of the elderly.

**Table 4:** Disparities of gender and urban–rural areas in the effect of widowhood on the risk of disability among older adults.

	(9)	(10)	(11)	(12)
	Male	Female	Rural	Urban
Widowhood	0.114***	0.030	0.040	0.120**
	(0.041)	(0.030)	(0.027)	(0.054)
Control variable	Yes	Yes	Yes	Yes
Constant	1.042**	2.051***	1.058***	2.594***
	(0.519)	(0.432)	(0.385)	(0.788)
*N*	5,141	6,631	9,095	2,677
*R* ^2^	0.046	0.054	0.047	0.053

*p < 0.10, **p < 0.05, ***p < 0.01.

**Table 5:** Disparities of educational attainment and age in the effect of widowhood on the risk of disability among older adults.

	(13)	(14)	(15)	(16)	(17)
	Non-literate	Illiterate	Aged 60–70	Aged 70–80	More than 80
Widowhood	0.054*	0.041	0.102**	0.033	0.005
	(0.030)	(0.047)	(0.040)	(0.045)	(0.115)
Control variable	Yes	Yes	Yes	Yes	Yes
Constant	0.522	2.163***	0.026	−0.064	−0.591*
	(0.547)	(0.581)	(0.151)	(0.120)	(0.354)
*N*	7,868	3,905	6,401	3,881	1712
*R* ^2^	0.035	0.077	0.026	0.038	0.128

*p < 0.10, **p < 0.05, ***p < 0.01.

### Channel analysis

The results in Table 6 show that in terms of health behavior, widowhood significantly reduced the sleep duration of the elderly compared to the marital status of married with spouse present. In terms of lifestyle, widowhood increased the probability of smoking among the elderly. There was an increasing trend in personal fitness expenditure after widowhood, but not significantly.

**Table 6:** Channel analysis of the effect of widowhood on the risk of disability among older adults.

	(18)	(19)	(20)
	Sleep duration	Smoking	Personal fitness expenditure
Widowhood	−0.196*	0.020**	0.009
	(0.119)	(0.010)	(0.121)
Control variable	Yes	Yes	Yes
Constant	11.555***	−0.186	3.748**
	(1.761)	(0.147)	(1.790)
*N*	14,536	10,394	14,448
*R* ^2^	0.005	0.009	0.021

*p < 0.10, **p < 0.05, ***p < 0.01.

### Moderating effect analysis

With the disappearance of spousal support after widowhood, the internal support from family and external social support are particularly important for elderly individual’s health in later life (23). Based on the immediate family, child financial support and contact frequency were selected as moderating variables and based on the external social support, the number of social activities in which the elderly participated was selected as the moderating variable to analyze the moderating effects of family and social support.

The results in Table 7 show that children’s financial support, frequency of children’s contact, and the number of social activities significantly reduced the risk of disability among the elderly after widowhood.

**Table 7:** Moderating effects of widowhood on the risk of disability among older adults.

	(21)	(22)	(23)
	Disability risk	Disability risk	Disability risk
Widowhood	0.077***	0.070***	0.077***
	(0.027)	(0.025)	(0.025)
Widowhood*Financial support from children	−0.004*		
	(0.002)		
Widowhood*Frequency of child contact		−0.002*	
		(0.001)	
Widowhood*Number of social activities			−0.033***
			(0.010)
Control variable	Yes	Yes	Yes
Constant	1.532***	1.547***	1.540***
	(0.337)	(0.337)	(0.337)
*N*	11,768	11,768	11,768
*R* ^2^	0.049	0.049	0.051

*p < 0.10, **p < 0.05, ***p < 0.01.

## Discussion

### Widowhood significantly increases the risk of disability

Firstly, based on the theory of the marital resource model, marriage has a health-protective effect, curbing health deterioration (7) and influencing the health behavior of older adults (8). With a living spouse, the spouse positively influences both health behavior management and health management environment of older adults (9). Married groups are more likely to have healthy lifestyles, good dietary habits, and do more exercise (10, 11). Secondly, the stress process model suggests that, with increasing age, the onset of illness and/or physical injury and the frequent death of relatives, especially generational peer deaths (12), gradually become important stressors, and being in a stress-exposed environment or having a lack of resources can have adverse effects on an individual’s physiological and psychological health (13).

### Heterogeneous effects of widowhood on risk of disability

There were disparities in the gender, urban–rural, education attainment, and age on the effect of widowhood on disability. From the perspective of gender differences, females tend to play more caregiving roles and are important providers of family support under differences in the division of household labor, and the death of elderly females can seriously affect care maintenance, thereby increasing “health shocks” and the risk of disability for elderly males. From the perspective of the urban–rural differences, for the rural elderly, there is a high proportion of traditional three-generation lineal household structures and, after widowhood, children who live together with the elderly can somewhat mitigate the negative effects of widowhood. For the urban elderly, on the other hand, the family model miniaturization emphasizes more the healthy role of the spouse and the negative impact of widowhood is more obvious (36). In terms of the level of education, educational level is associated with better economic status which thus makes elderly individuals more likely to choose to spend their later years away from their children, thus affecting the risk of disability after widowhood. Regarding age, widowhood leads to the significantly higher risk of disability among the elderly aged 60–70 years, which is also in line with the study (37).

### Channel analysis

Widowhood significantly reduced the sleep duration and increased the probability of smoking among elderly. Possible explanations are that, on the one hand, based on the stress perspective, widowhood status may increase psychological stress in older adults, such as depression, loneliness, and other negative emotions; furthermore, stress affects the elderly’s health management behaviors, which leads to a decrease in sleep time and the emergence of smoking behaviors. On the other hand, based on the marriage resource model, after widowhood, the health protection role of the spouse disappears, the elderly’s own health awareness decreases, and poor lifestyles emerge, such as less sleep and more smoking. Possible explanations for the lack of significant impact of widowhood on health care costs are that, while widowhood stress deteriorates the health levels and promotes the health care expenditure, the strain on household resources leads to a decrease in health investment, thus making it difficult to estimate the significant effect of widowhood on health care expenditure.

### Moderating effect analysis

Financial support from offspring, frequent contact from children, and more participations in social activities significantly reduced the disability risk among the elderly after widowhood. From the stress process theory, the financial support of children, the frequency of children’s contact, and the number of social activities put individuals in different stress-exposure environments (38) and stress vulnerability: the financial support from offspring can enhance individuals’ economic resources and reduce their stress vulnerability; the increase in the frequency of children’s contact and the number of social activities can reduce loneliness and put individuals in a less stress-exposed environment, thus alleviating the impact of widowhood on their physiological and psychological well-being.

## Limitations

Despite the notable findings, this study, like most others, had limitations. Firstly, there were long-term and short-term differences in widowhood status, but the study in this paper could not discern the effect of the duration of widowhood status on the risk of disablement. Secondly, due to the limitation of database data, this paper did not provide an accurate assessment of stress. This paper attempted to add stress-related factors as indicators that could reflect the level of stress, but this was still not precise enough. Finally, the survey sample used in this paper covered almost the entire country, so there was an impact of the uneven development between regions on the results. This paper ignored the impact of regional differences in economic level, medical level, and social support services on the risk of disability.

### Strengths

Firstly, this paper examined this issue based on a social context with Chinese characteristics, providing an examination of data from a large sample. Secondly, the robustness test of this paper was strong, which was conducted here by replacing the data sample, the disability measurement index, and the analysis method. Thirdly, this paper found that family and social support was an effective way to reduce the risk of disability in the widowed elderly group, which has some positive practical implications for improving health in later life.

## Conclusion

Based on the stress process model theory and the data from the China Health and Retirement Longitudinal Study (CHARLS) in 2011, 2013, 2015, and 2018, this study explored the impact of widowhood on the risk of later-life disability in the elderly in China and the heterogeneous performance in different older age groups, and then investigated the impact channels and regulation. The main research findings are as follows:

First, widowhood significantly increases the risk of disability among elderly people compared to being married with a spouse present. These results remain robust after further changes to the data sample, measuring indicators, and regression methods. Second, there was heterogeneity in the effect of widowhood on the risk of disability in older adults. The widowhood effect is particularly pronounced in the male, urban, educated, and 60-to-70-year-old age group. Third, the channels of the impact of widowhood on the risk of disability in older adults can be categorized as psychological stress and health behaviors; this was evidenced by a decrease in sleep duration and an increase in the probability of smoking in older adults who were widowed. Fourth, financial support and spiritual comfort from children within the family and external social support can significantly reduce the risk of disability and mitigate the negative health shocks of widowhood in older adults.

The research implications of the above findings are the following: first, widowhood leads to the disappearance of health concerns from the spouse, and the reduced health importance of the widowed group requires active health interventions from society, with especially the male, urban, educated, and 60-to-70-year-old age group being the priority population for health interventions. Second, society encourages and guides children to provide financial support and companionship to the bereaved elderly by actively promoting the traditional Chinese virtue of filial piety, thereby reducing the risk of disability for the bereaved elderly. Third, the social support is more likely to be given to the bereaved elderly group, and the bereaved elderly group is actively encouraged to participate in community activities. By participating in social activities to build a new social support network, the bereaved elderly can fill the loneliness generated by widowhood and gain more psychological companionship and social acceptance, thus reducing their own health risks.

## Data availability statement

The original contributions presented in the study are included in the article/supplementary material, further inquiries can be directed to the corresponding author.

## Author contributions

YW made a substantial contribution to the concept and design of the article in the writing and empirical analysis. JP and SX collected and analyzed the data, and drafted the article. YW and JP collected relevant literature and data, adjusted the article format, and carefully revised and polished the language. All authors contributed to the article and approved the submitted version.

## Conflict of interest

The authors declare that the research was conducted in the absence of any commercial or financial relationships that could be construed as a potential conflict of interest.

## Publisher’s note

All claims expressed in this article are solely those of the authors and do not necessarily represent those of their affiliated organizations, or those of the publisher, the editors and the reviewers. Any product that may be evaluated in this article, or claim that may be made by its manufacturer, is not guaranteed or endorsed by the publisher.

## Footnotes

^1^It is worth clarifying that household registration is not living area. Household registration is an existing system unique to the historical context of China. Since 1955, when China established a system to separately manage the agricultural and non-agricultural populations, China’s long-standing household registration system, characterized by a binary division between urban and rural areas, has led to different states of urban and rural development.

^2^Personal fitness expenditure data were derived by dividing household spending on fitness expenditure (including fitness exercise and product equipment, health care products, etc.) in the past year by the number of people in the household.
